# How different is different? Systematically identifying distribution shifts and their impacts in NER datasets

**DOI:** 10.1007/s10579-024-09754-8

**Published:** 2024-07-18

**Authors:** Xue Li, Paul Groth

**Affiliations:** Informatics Institute, University of Amsterdam, Science Park, Amsterdam, 1098 XH Netherlands

**Keywords:** Distribution shift, Named entity recognition

## Abstract

When processing natural language, we are frequently confronted with the problem of distribution shift. For example, using a model trained on a news corpus to subsequently process legal text exhibits reduced performance. While this problem is well-known, to this point, there has not been a systematic study of detecting shifts and investigating the impact shifts have on model performance for NLP tasks. Therefore, in this paper, we detect and measure two types of distribution shift, across three different representations, for 12 benchmark Named Entity Recognition datasets. We show that both input shift and label shift can lead to dramatic performance degradation. For example, fine-tuning on a wide spectrum dataset (OntoNotes) and testing on an email dataset (CEREC) that shares labels leads to a 63-points drop in F1 performance. Overall, our results indicate that the measurement of distribution shift can provide guidance to the amount of data needed for fine-tuning and whether or not a model can be used “off-the-shelf” without subsequent fine-tuning. Finally, our results show that shift measurement can play an important role in NLP model pipeline definition.

## Introduction

Differences between training and inference distributions are a common occurrence in the field of Natural Language Processing (NLP). This difference can be observed, for instance, when the input data undergoes changes over time or when a model is employed on data from a new domain. This is known as *distribution shift* (Quionero et al., 2009). Consider the following example from Named Entity Recognition (NER):

**Fig. 1:**
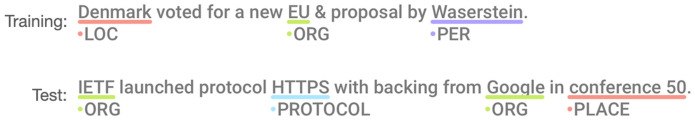
An example of differences in sentences that would be affected by a shift in the distribution of the NER training data

**Fig. 2:**
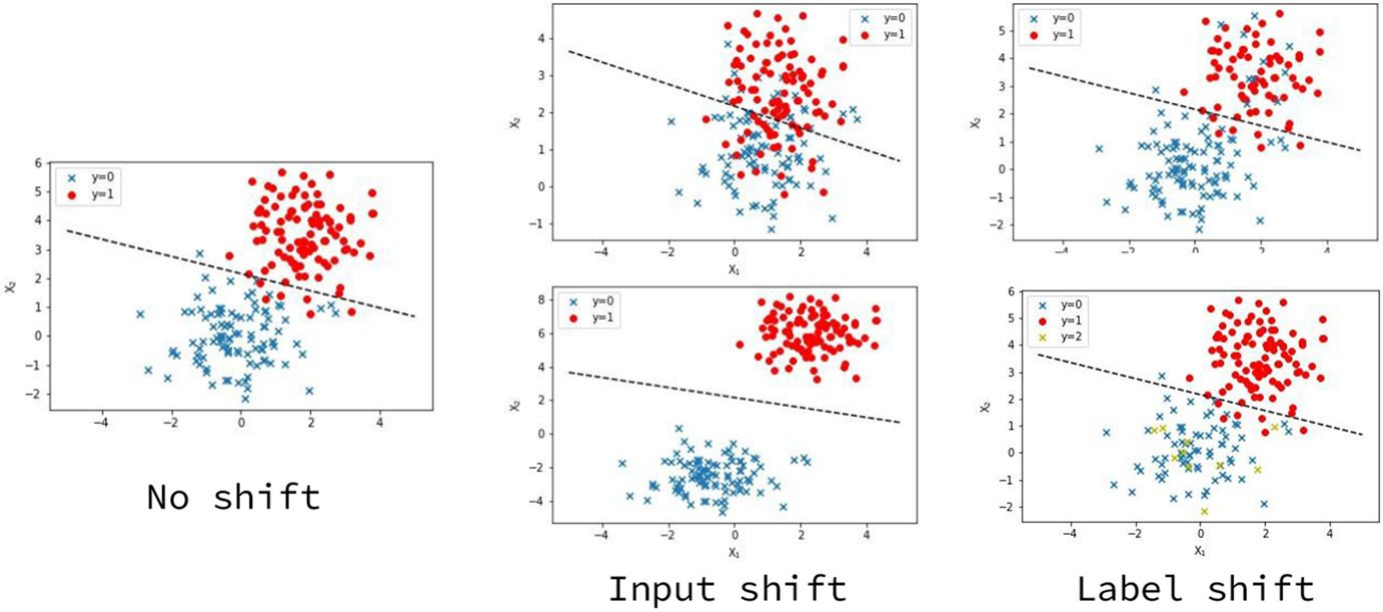
Toy examples of input shift and label shift. The dashed lines indicate an existing machine-learning classifier that performs well at training time. We show two possible scenarios when the relationship of x and y changes for each type of shift

The example shows two phenomena (Fig. 1). First, the entities in the training example tend to be relatively well-known entities (e.g. EU), which are highly probable to be present in the data sources utilized by pre-trained language models (Devlin et al., 2019; Lee et al., 2019) that are widely used for NLP tasks. Conversely, the entities in the inference example are unique to a particular domain (e.g. IETF). Second, the labels in the training example differ from the ones in the inference example. This is because entities from different domains possess different types, such as “Organization" versus “Protocol", and variations in labelling for the same type, such as “Location" and "Place". These phenomena represent two common shifts in NLP: input distribution shifts and label distribution shifts (Quionero et al., 2009). We show how these two types of shifts can affect the performance of an existing classifier with a toy example in  Fig. 2. The example shows situations when the test distribution differs from the training distribution, often caused by the change of the underlying relationship between the input *x* and the label *y*. When shifts happen, often the performance of the pre-trained classifier (shown in dashed lines) will no longer hold. In this work we primarily focus on category shift in the label space (Lekhtman et al., 2021). Despite the substantial body of literature on measuring domain similarity (Dai et al., 2019), detecting *when* a shift occurs remains a challenging task in the field. This task is known as *shift detection*.

A key area where shift detection is useful is *domain adaptation*, which aims at adapting a model in the presence of distribution shifts (Csurka, 2017). One of the common supervised approaches to achieve adaptation is fine-tuning deep neural networks (Vu et al., 2020). While fine-tuning can be effective, there is still a cost, such as determining the required amount of additional data for fine-tuning. To inform this decision, shift detection methods are frequently employed in other areas that employ machine learning (Rabanser et al., 2019; Cobb & Van Looveren, 2022; Kulinski et al., 2020). This work frequently adopts statistical hypothesis testing as an underlying principled approach to the problem (Rabanser et al., 2019; Cobb & Van Looveren, 2022). Statistical two-sample testing is a methodology for determining whether the distribution of the training data *p* is equivalent to the distribution of the test data *q*. While this approach has been explored for computer vision tasks involving high-dimensional data, it has seen limited application to NLP

Hence, to better inform these decisions and quantify the potential impact of distribution shifts, this paper performs a systematic investigation of shifts across benchmark corpora using statistical tests, which have been widely adopted for shift detection in the context of other machine learning tasks. In this work, we specifically focus on the NER task and detect distribution shifts across 12 different datasets that are representative of various domains. We use word frequency and sentence-level representations to characterize input distributions, and label frequency to characterize label distributions. Appropriate statistical tests are identified for each representation and employed to detect and quantify shifts. We then investigate the impact of domain shift in both the input and label space on performance in the supervised setting. We establish a relationship between the shift distance and the performance degradation. These results provide insights into what statistical test one needs to perform to make such a determination.

Summarizing, the contributions of this paper are as follows:

- The systematic measurement of distribution shift between 12 NER benchmark datasets covering multiple domains.
- Systematic measurement of how much a distribution shift impacts performance for NER, a prototypical NLP task.
- Evidence that sentence-based representations provide better information for shift detection for the NER task.

## Related work

Distribution shifts are prominent in real-world applications (Specia et al., 2020; Michel, 2021; Recht et al., 2019; Engstrom et al., 2019), leading to growing interest in detecting them for machine learning tasks (Rabanser et al., 2019; Cobb & Van Looveren, 2022; Kulinski et al., 2020).

***Shift types*** In the broader landscape of machine learning, Wiles et al. (2022) conducted a fine-grained analysis of distribution shifts, classifying them as spurious correlation, low data drift, and unseen data shift. Additionally, they evaluated 19 different methods on both synthetic and real-world datasets for vision tasks.

***The Use of Statistical Tests*** The use of statistical tests for dataset shift detection was brought to the fore by Rabanser et al. (2019). In their work, they developed a dataset shift detection framework which contains a dimensionality reduction component and a two-sample-testing component. They investigated multiple combinations of methods for each component, and tested on artificially generated covariates and label distribution shifts. Recently, based on two-sample tests for shift detection, Cobb & Van Looveren (2022) developed a general drift detection framework borrowing machinery from causal inference. The framework is used to deal with the situation when the inference data are not expected to form an i.i.d. sample from the historical data distribution.

***Domain similarity*** Within the field of NLP, researchers have explored various methods for measuring domain similarity in the context of domain adaptation including using target vocabulary covered rate and language model perplexity(Dai et al., 2019). However, these methods work well under the assumption that there are sufficient data from the source and target distribution. Therefore, in our work we adopt non-parametric statistical hypothesis testing framework to detect shift without knowing the actual parameters of the population.

***Shift detection in NLP*** Within NLP, Arora et al. (2021) focused on out-of-distribution texts and two approaches for detection. Shifts are categorized into *background* shift and *semantic* shift. Model calibration and density estimation are investigated for shift detection across 14 pairs of natural language understanding datasets. Comparing density estimation methods and calibration methods. We investigate different types of shifts than these works.

Given the importance of shift detection, a number of datasets have been developed (Koh et al., 2021; Malinin et al., 2022), however, they are not for the widely used task of NER.

Our work adds to this existing literature. First, we employ widely used labelled NER datasets and compare not only changes in fields (e.g. science to finance) but also changes in text style (e.g. news style text to social media style text). Second, we test the impact of representation choice on shift detection. Lastly, we provide new evidence for the performance impact of distribution shifts on task performance.

**Table 1:** List of annotated datasets for English NER from different domains

Corpus	Year	Document source	Domain	# Types	Category
GUM	2017	Wiki-family	Various	11	Wiki data
Wikigold	2009	Wikipedia text	Various	4	Wiki data
BTC	2016	Twitter	Mainstream news	3	Informal text
W-NUT17	2017	User-generated text	Various	6	Informal text
CEREC	2005	Informal emails	Work	4	informal text
AnEM	2012	Anatomical text	Anatomy	11	Specific field
i2b2-06	2006	Clinical text	Biomedical	7	Specific field
SEC-Filings	2015	Electronic filings	Finance	4	Specific field
SciERC	2018	Scientific abstracts	Scientific	6	Specific field
Re3d	2018	Documents related to defense and security analysis	Conflict in Syria and Iraq	10	Specific field
CoNLL-03	2003	Reuters news	Mainstream news	4	News
OntoNotes	2007-2012	Magazine, news, web, tele, etc	Various	18	General

## Methodology

Our methodology consists of the following steps: data collection; representation choice; statistical hypothesis testing and shift impact measurement. For data collection, we acquire datasets from different domains. Domains are characterized by their language usage arising from the style employed to the use of language particular to given field usage. For all datasets, both the space of input text and the space of labels are considered. In terms of representations, two types of representations are used for the input and one for labels. Statistical hypothesis testing appropriate for each representation is used to detect distribution shifts. The calculated statistics are then used to measure the extent of a shift. Lastly, the impact of each shift on model performance is determined. We now walk through each of these steps in detail.

### Data collection

We collected 12 datasets from different domains covering news, social media, encyclopedic content, finance, science, emails, and business. Table 1 shows the list of datasets with the published year, document source, domains and the number of entity types. Table 2 shows the list of datasets and their entity types. We group the datasets into five categories, which we now describe in-turn.

**Table 2:** List of NER datasets with corresponding entity types

Corpus	Data size	# Types	Entity types
GUM	3,424	11	Organization, Person, Location, Event, Abstract, Object, Time, Substance, Plant, Quantity, Animal
Wikigold	1,688	4	Organization, Person, Location, Miscellaneous
BTC	9,318	3	Organization, Person, Location
W-NUT17	5,591	6	Organization, Person, Location, Group, Product, Creativework
CEREC	2,031	4	Organization, Person, Location, Digits
AnEM	4,423	11	Multi-tissue_structure, Organism_substance, Organism_subdivision, Organ, Cellular_component, Cell, Immaterial_anatomical_entity, Tissue, Pathological_formation, Anatomical_system, Developing_anatomical_structure
i2b2-06	40,280	7	Person, Location, ID, Date, Phone, Age
SEC-Filings	1,435	4	Organization, Person, Location, Miscellaneous
SciERC	2,687	6	Material, OtherScientificTerm, Generic, Method, Task, Metric
Re3d	948	10	Organization, Person, Location, Temporal, Nationality, Quantity, Weapon, Money, MilitaryPlatform, DocumentReference
CoNLL-03	17,350	4	Organization, Person, Location, Miscellaneous
OntoNotes	17,760	18	Organization, Location, Person, Work_of_Art, Cardinal, Event, NORP, Date, FAC, Quantity, Ordinal, Time, Product, Percent, Money, Law, Language

Data size represents the number of sentences in each dataset

#### Wiki data

**GUM** (Zeldes, 2017) (the Georgetown University Multilayer Corpus) is collected and expanded as part of the curriculum of a course. The current corpus contains texts from public wikis (e.g. Wikinews, Wikivoyage, wikiHow, Wikipedia) as well as social media sites (e.g. Reddit, Youtube). Example types include *event*, *time*, *animal* and *abstract*. **Wikigold** (Balasuriya et al., 2009) is a gold-standard NER dataset sourced from Wikipedia. Wikigold has standard types such as *person* and *organization*.

#### Informal text

Formal texts such as in news and Wikipedia are normally verified by multiple people sometimes even experts. Hence, the majority of text has correct grammar and spelling. In comparison, user-generated informal data such as social media texts, often contain less formal language usage characterized by slang, poor grammar, misspellings, the use of satire, etc. **BTC** (Derczynski et al., 2016)(Broad Twitter Corpus) is a NER dataset where the source data is from Twitter that not only has tweets on general topics but also on specific topics such as disasters. BTC includes 3 types: *person*, *location* and *organization*. **WNUT17** (Derczynski et al., 2017) is a NER dataset where the text sources are Reddit, Twitter, YouTube and StackExchange comments. WNUT17 focuses especially on emerging and rare entities. The dataset contains 6 types, including *creative*, *corporation* and *product* besides common types. **CEREC** (Dakle et al., 2020) is a large-scale corpus for entity resolution in email conversations. The emails are taken from the first large public corpus the Enron Email Corpus (Klimt & Yang, 2004) which contains emails of 150 employees of the Enron Corporation. Cerec contains standard types such as *person* and *digits* type.

#### Specific fields

**AnEm** (Ohta et al., 2012) is a corpus annotated with species-independent anatomical entity mentions. The texts are from academic papers from the biomedical scientific literature. AnEm contains 11 domain-specific types such as *organ*, *cell* and *organism_substance*. **i2b2** (Uzuner, ö., Luo, Y., Szolovits, P., 2007) is a corpus that contains unstructured clinical notes from the Research Patient Data Registry at Partners Healthcare. The dataset consists of 8 types such as *hospital*, *phone* and *doctor*. **SEC-filings** (Salinas Alvarado et al., 2015) (U.S. Security and Exchange Commission filings) is a randomly selected and manually annotated finance dataset. The texts are from public-domain financial reports. The dataset includes standard types from CoNLL, i.e. *organization*, *person*, *location* and *misc*. **SciERC** (Luan et al., 2018) is a dataset that includes annotations for scientific entities in 500 scientific abstracts from AI conferences and workshop proceedings. The dataset focuses on scientific related types including *material*, *method* and *task*. **Re3d** (Dstl & Laboratory, n.d.) was constructed from documents that are relevant to the defence and security analysis domain, specifically, focusing on the topic of the conflict in Syria and Iraq. It includes domain-specific types such as *weapons* and *military platform*.

#### News

**CoNLL-03** (Tjong Kim Sang & De Meulder, 2003)^1^ is a dataset where the texts are taken from the Reuters news stories from 1996 to 1997. It contains the standard types including *person*, *location*, *organization* and *misc*.

#### General

**OntoNotes** (Weischedel et al., 2017)^2^ is a large annotated corpus that consists of various genres of texts including news, conversational telephone speech, weblogs, newsgroups, broadcast, and talk shows). OntoNotes include a large variety of types (18) including common types and less common ones such as *money* and *percent*.

Even though the datasets are grouped into five categories, there is still overlap. Wiki-based datasets and OntoNotes or CoNLL belong to different categories, but they might share many similar general entities. This is because common entities in the news are highly likely to have Wikipedia pages. Intuitively, the “similarity” between datasets in the wiki group should be larger. Conversely, the “similarity” between the domain-specific financial dataset SEC and the news dataset CoNLL should be smaller. We introduce methods to statistically quantify the distance between datasets in the following sections.

For all datasets, we preprocess them as follows. Duplicates are removed to prevent overfitting. Labels are unified across datasets shown in Appendix A. Different datasets use different labels to refer to the same type. Hence, to better compare performance, we unify the labels with the same semantic meanings. For example, ‘person’ and ‘PER’ will be unified under the same label.

### Shift detection and measurement

We use statistical testing to determine and measure shifts between datasets. Formally, given a labeled source domain data $\{({\varvec{x}}_1, {\varvec{y}}_1),\ldots , ({\varvec{x}}_n, {\varvec{y}}_n)\} \sim p$ and labeled target domain data $\{(\varvec{x'}_1, \varvec{y'}_1),\ldots , (\varvec{x'}_n, \varvec{y'}_n)\} \sim q$, shift detection determines whether *p* equals *q*. The null hypothesis is $H_0: p = q$ and the alternative hypothesis $H_0: p \ne q$. The statistical values are used as shift measurements. Both shifts occurring in the input distribution $p({\varvec{x}})$ and the label distribution $p({\varvec{y}})$ are investigated.

When forming the dataset pairs for the hypothesis testing, the distance functions we use are bi-directional, meaning that given a function *Dist* calculating distance, *Dist(p, q)* = *Dist(q, p)*. Therefore, we measure the distribution shifts using combinations without repetition. Additionally, we include the distance of each dataset to itself as a sanity check. This approach results in 66 unique combinations plus 12 self-comparisons, in a total of 78 pairs.

We now discuss the representations we use for the datasets and the corresponding statistical tests we employ.

#### Representation for input space

We investigate two different representations for the input space.

Word frequencies: in this setting, ${\varvec{x}}$ represents the frequency of each word. The underlying assumption is that the occurrences of words within a dataset indicate how important a word is. The word frequency distribution over the vocabulary represents the dataset.

Distributional representation: In this setting, each instance of ${\varvec{x}}$ is an n-dimensional vector representation of a sentence within a dataset. Sentence-BERT (Reimers & Gurevych, 2019) is used to encode each sentence. The idea behind sentence-BERT is that semantically similar sentences are closer in vector space (Reimers & Gurevych, 2019). The data points in this n-dimensional space are the distribution for each dataset.

#### Representation for label space

Within the NER task, datasets from different domains have different types of entities. We use category counts as our label distribution $p({\varvec{y}})$. Among different domains, the most general types include Person, Organization, Location and Miscellaneous. As mentioned in subsection 3.1, we unify labels with the same semantic meanings. We note that very field-specific datasets will have a different label space than more general datasets.

Following recent work on distribution shifts, for label space, we formulate the problem as one of *unseen data shift* where some attribute values are unseen under *p* but are seen under *q* (Wiles et al., 2022). For example, the type *Method* might have zero observation in many datasets such as in CoNLL and Wikigold, but it will have many observations in dataset SciERC. However, it does not necessarily mean that there are no entities that have the type Methods in the Wikigold dataset, but due to specific data generation processes, those entities are not annotated. We see this as an outcome of different sampling processes. We assume all datasets share a common label set ${\varvec{A}}^l$ and some labels in the set are unseen in *p* but are seen in *q* due to systematic sampling error.

#### Statistical hypothesis testing

For each type of representation, a different statistical test is necessary, which we now detail. Shift decisions are reported based on the significant level. By default, we use 0.05 as the significant threshold for all tests. Furthermore, we use this statistical testing as a means to measure distribution shift and draw a connection between shift and performance.

***Chi-Squared Test*** For frequency distributions of input and label count distribution, each sample ${\varvec{x}}_n$ is one categorical value that represents word occurrence in the domain. We adopt Pearson’s Chi-Squared test, a parametric test for determining if two frequency distributions are the same. The crucial underlying assumption is that a corpus is modelled as a sequence of independent Bernoulli trials. The relevant statistic $\chi ^2$ can be computed as:

$$\begin{aligned} \chi ^2 = \sum _{i=1}^2 \sum _{j=1}^C \frac{(O_{ij} - E_{ij})^2}{E_{ij}}, \end{aligned}$$

where $O_i$ is the observed value for category i and $E_i$ is the expected value for category i. All word occurrences below 5 are filtered out.

There has been a long debate if the chi-squared test, or statistical testing in general, should be applied for corpus linguistics (Gries, 2005). However, it is still widely employed within the literature (Rabanser et al., 2019). Given that the distribution shift literature also employs chi-squared testing, we also make use of it here.

We employ two data processing procedures while using Chi-Squared tests. First, before applying the Chi-Squared test to data, we implement a normalization procedure to ensure that both the observed and expected values are on the same scale (Underhill & Bradfield, 2013). This normalization enhances the robustness of the test to different sample sizes. Second, by design, label distribution may contain a considerable number of zeros for certain categories. Since Chi-Squared test is not viable when dividing by zero, we added a small constant ($1e-5$) to each category to ensure that we obtain results without changing the numerical meaning of the results.^3^

Another potential test for this sort of distribution is the Kolmogorov-Smirnov (KS) two-sample test. However, this test fits the cumulative distribution which requires values to be sorted. Sorting items in a vocabulary is not meaningful.

***Maximum Mean Discrepancy (MMD)*** For multi-dimensional representations obtained from sentence-BERT, we employ MMD (Gretton et al., 2012), a nonparametric kernel-based two-sample test to determine if two samples are drawn from two different distributions *p* and *q*. MMD tries to calculate the $L_2$ distance between the mean embeddings $\mu _p$ and $\mu _q$ of the distributions in a reproducing kernel Hilbert space ${\mathcal {F}}$ as:

$$\begin{aligned} \textbf{MMD}^2(P, Q) =<\mu _p, \mu _p> - 2<\mu _p, \mu _q> + <\mu _q, \mu _q>. \end{aligned}$$

Empirically, we use the unbiased estimate of the squared MMD statistic:

$$\begin{aligned} \textbf{MMD}^2 = \frac{1}{m^2 - m} \sum _{i=1}^m \sum _{j\ne i}^m \kappa ({\varvec{x}}_i, {\varvec{x}}_j) + \frac{1}{n^2-n} \sum _{i=1}^n \sum _{j\ne i}^n \kappa ({\varvec{x}}'_i, {\varvec{x}}'_j) - \frac{2}{mn} \sum _{i=1}^m \sum _{j=1}^n \kappa ({\varvec{x}}_i, {\varvec{x}}'_j) \end{aligned}$$

where the kernel is computed with a squared exponential function $\kappa ({\varvec{x}}, \tilde{{\varvec{x}}})=e^{-\frac{1}{\sigma }|{\varvec{x}} - \tilde{{\varvec{x}}} |^ 2}$. $\sigma$ is the median distance between points (Gretton et al., 2012).

### Impact on performance

The last step in our method is to detect how the shift affects model performance. Our hypothesis is that *as the degree of distribution shift increases, so does the likelihood that a model makes an error and hence the degree of this error will also increase.* As one of the most widely used pre-trained language models, we use BERT (Devlin et al., 2019) to measure performance. Specifically, we measure the effect of shifts from *p*(*x*) and shifts from *p*(*y*) on performance.

We fine-tune the BERT model on one dataset and then test its performance across all datasets to identify any performance degradation. When adapting BERT for NER, we treat it as a sequence-to-sequence task utilizing the BIO tagging scheme where each token in a sentence is tagged with Begin, Inside, or Outside to indicate named entity boundaries. Then, we add one fully-connected dense layer for predicting tags. A cross-entropy loss is used for calculating the loss between predicted tag sequence and the golden tag sequence.

Specifically, we first split each dataset into a training set and an inference set, ensuring that all models trained on one dataset can also be tested on the same dataset. We follow the classic machine learning split ratio of 80:20, training on 80% of the dataset and testing on 20%. We then pair any two of the datasets and use the training set of the first as the source domain and the inference set for the second as the target domain. We fine-tune the original baseline model on the source training set and evaluate on the target inference set. Fine-tuning is performed for 10 epochs. Similar to the original BERT paper, we use a batch size of 32 and a learning rate of 5e-5. To ensure robustness in our results, we report the average performance across five trials, each with different random samplings. We train and test our model on GPU GeForce 1080Ti with 11GB GPU RAM. The fine-tuned BERT model’s architecture consists of 12 layers of attention blocks, with each layer having a hidden size of 768 in the embedding layers. We follow the standard configuration of the BERT-base model, which comprises approximately 110 million parameters (Devlin et al., 2019). When we finetune it for NER task, we add one more fullyconnected dense layer. The number of parameters for each neuron is hidden size + bias (768 + 1) = 769. The final number of parameters added is $num\_tags$ * 768 $\approx$ 15,380 parameters. Our code for both the hypothesis testing and evaluation is available as supplementary material.

To draw a connection between distribution shifts and performance degradation, we calculate the correlation between the measurement *shift* and the performance difference $perf_{ab}$ between any two datasets *a* and *b*.

### Experimental setup

We conduct various experiments under different setups. For shift detection, we verify the validity of the tests on the sampled datasets of the same corpus. If the results indicate no shift, this implies that the testing has effectively validated that the two distributions are the same. We subsequently apply tests to all pairs of datasets.

As illustrated in Table 2, the datasets exhibit varying sizes. In general, ML models perform better when fine-tuned with more data. To mitigate the potential biases derived from data size, we uniformly sample a subset of 948 samples from each dataset and perform all experiments. This is because the minimum number of samples among all datasets is 948 from the Re3d dataset. In practice, engineers often fine-tune the model on the full dataset for better performance. To investigate the correlation under this scenario, we also employ identical tests and performance measures on the original full-sized datasets, the results of which are included in the Appendix A.

For the performance measures, BERT is pre-trained on a particular scope of texts and may favour datasets from certain domains. To address this potential bias, we utilize both BERT-base and BioBERT-base (Lee et al., 2019) and compare their respective performance outcomes. The complete results are provided in the Appendix B.

**Fig. 3:**
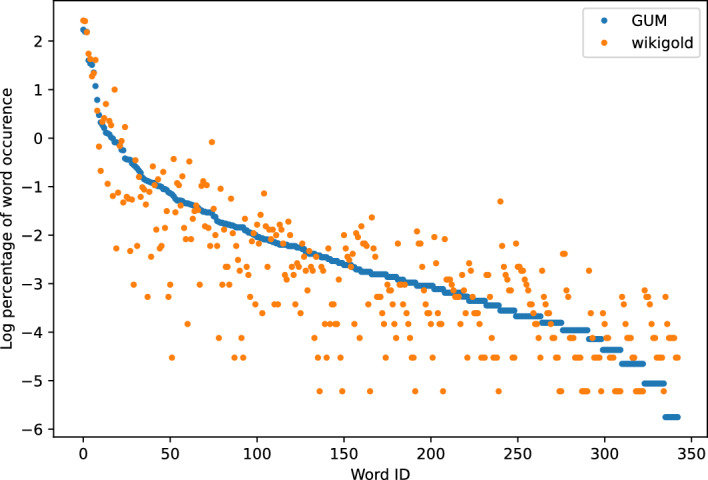
GUM and Wikigold

**Fig. 4:**
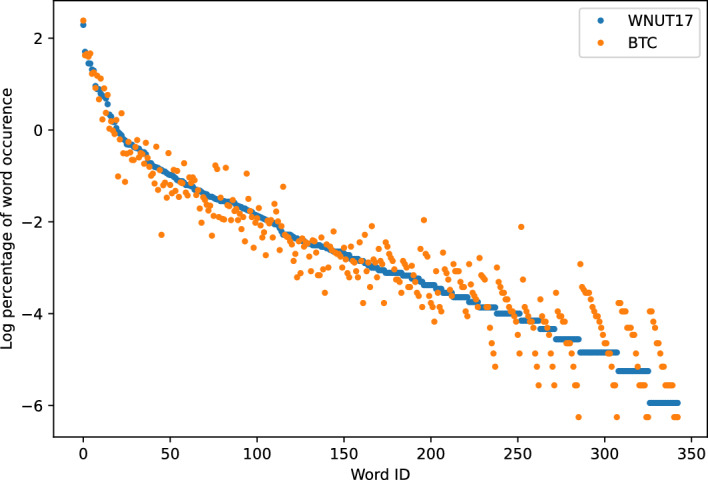
WNUT-17 and BTC

**Fig. 5:**
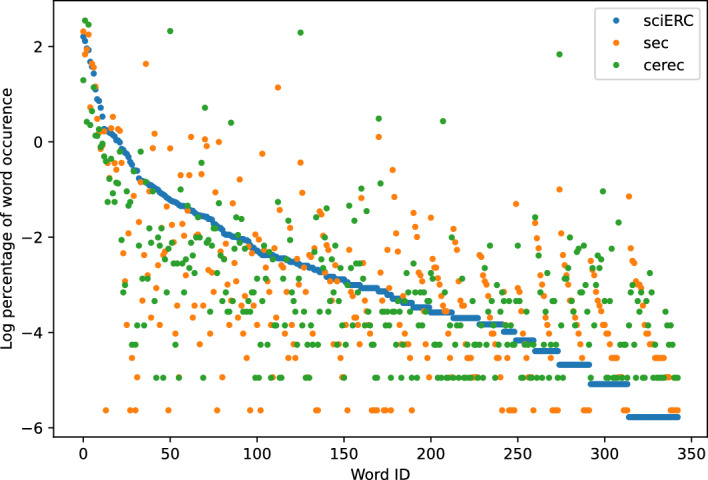
SciERC, SEC and CEREC

**Fig. 6:**
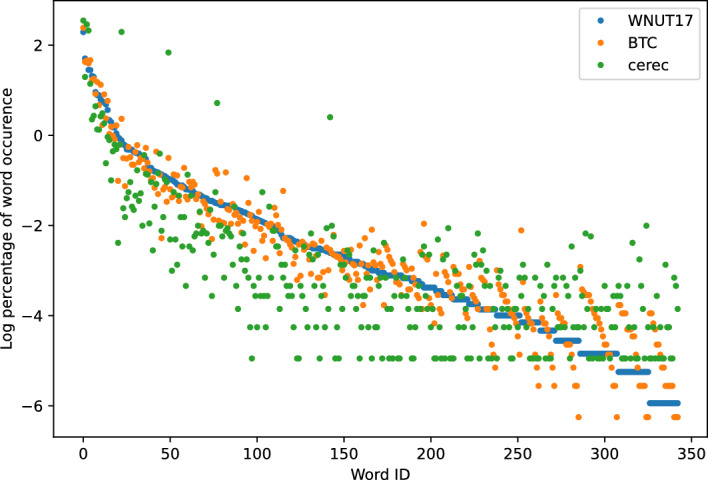
WNUT-17, BTC and CEREC

**Table 3:** MMD statistics for **input embedding distribution** on full-sized datasets with a different number of samples

Source data	Target data	Number of samples from test					
		5	50	200	500	948	2000
conll	conll	− 0.30	− 0.03	− 0.01	− 0.0029	− 0.0015	− 0.0007
cerec	cerec	− 0.29	− 0.03	− 0.01	− 0.0029	− 0.0015	− 0.0007
BTC	WNUT17	0.02	0.01*	0.01*	0.01*	0.01*	0.01*
GUM	wikigold	0.00	0.02*	0.02*	0.02*	0.02*	0.02*
conll	wikigold	0.03*	0.04*	0.03*	0.03*	0.03*	0.03*
ontonotes	GUM	0.02	0.02*	0.04*	0.03*	0.03*	0.03*
GUM	WNUT17	0.03	0.04*	0.03*	0.03*	0.03*	0.03*
.	.	.	.	.	.	.	.
BTC	SEC	0.10*	0.13*	0.15*	0.15*	0.14*	0.15*
WNUT17	SEC	0.16*	0.14*	0.14*	0.14*	0.14*	0.15*
i2b2-06	SEC	0.17*	0.16*	0.16*	0.15*	0.15*	0.15*
SEC	sciERC	0.11*	0.13*	0.15*	0.16*	0.15*	0.15*
re3d	SEC	0.20*	0.15*	0.15*	0.15*	0.16*	0.15*

This is ordered by the distance between pairs of datasets with 2000 samples. Sign $\star$ indicates there is a shift detected. The full result is in Table 9

## Results and discussion

We now present the results of applying the method detailed above. We begin with an analysis of the input datasets to verify our hypothesis about the shift between distributions representing shift between domains.

### Datasets analysis

Figures 3, 4, 5, 6 show the word frequency plots on selected pairs of datasets. WNUT-17 and BTC both include text from Twitter, and we see that word frequency is similar across both datasets. Conversely, in the case of SciERC, SEC and Cerec datasets, which more distinctly represent different domains, we observe greater dispersion within their respective word frequency distributions. Intuitively, these results suggest that word frequency distributions can serve as an indicator of a domain.

### Hypothesis testing

Considering space limitations, Tables 3, 4, 5 display only a subset of results for hypothesis testing with corresponding distributions. We selected two dataset pairs wherein the source and target distribution are from the same dataset. Then we selected the top 5 and bottom 5 pairs in ascending order. All tables are selected following the same style. All tests are conducted on both sampled datasets and full datasets, and complete results for all dataset pairs are available in Appendix B.

#### Chi-squared testing for input distribution

Table 4 shows the chi-squared testing on both sampled dataset pairs and original-sized dataset pairs. For Chi-squared tests, the shift is made based on the p-value of the testing. A common cutoff for rejecting the null hypothesis in this context is a p-value less than 0.05, indicating a statistically significant difference in distributions. The results of the Chi-squared tests are provided as a proximity for the distribution differences. The higher the value, the greater the disparity. The distance between the same distribution is also reported as a sanity check. When the source and target distributions are equivalent, the testing indicates that no shift is detected. This indicates that the testing is capable of identifying when two distributions are identical.

For the full-sized datasets, the left table in Table 4 reveals that among the 78 dataset pairs, 13 pairs are detected with shifts. Meanwhile, the right table indicates that for the sampled datasets, out of the same 78 pairs, 22 pairs are detected to have shifts. These results suggest that the test is more sensitive to identifying shifts when there is a smaller sample size. On closer inspection of the dataset pairs, we observe that out of the 13 shift-detected full-sized pairs, 11 pairs are also detected in the sampled datasets, which reaches an approximately 84.6% agreement. Additionally, the full results presented in Table 4 reveal that a higher Chi-Squared value does not necessarily imply the detection of a shift. For instance, while the OntoNotes and i2b2 datasets have high Chi-Squared values, no shift is detected. This outcome could arise due to the data samples being non-representative of the full distribution, thereby resulting in the test’s inability to make a confident conclusion.

Analyzing these results, we note that BTC and WNUT-17 datasets have the smallest distance, which is inline with the frequency plots in Fig. 4. On the other hand, the BTC dataset and SEC finance dataset have the furthest distance, which, as expected, reflects that these two datasets have very different text styles. One surprising outcome is GUM and SciERC which have a relatively small distance using this representation while being from what appear to be different domains. These examples illustrate that this test can quantify the distance between datasets (Fig. 10).

**Table 4:** Chi-squared statistics for input frequency distribution on full-sized datasets (left) and sampled datasets (948 samples)(right) in ascending order

Source data	Target data	Statistics	Shift decision	Source data	Target data	Statistics	Shift decision
conll	conll	0.00		conll	conll	0.00	
cerec	cerec	0.00		cerec	cerec	0.00	
BTC	WNUT17	96.04		GUM	BTC	75.66	
GUM	WNUT17	123.53		GUM	WNUT17	81.64	
GUM	BTC	127.48		BTC	WNUT17	93.89	
ontonotes	WNUT17	144.48		ontonotes	WNUT17	98.60	
ontonotes	re3d	149.69		ontonotes	BTC	124.59	
.	.	.	.	.	.	.	.
cerec	ontonotes	4,726.86	$\clubsuit$	WNUT17	sec	2,590.02	$\clubsuit$
conll	sciERC	6,930.89	$\clubsuit$	ontonotes	sciERC	2,608.52	$\clubsuit$
cerec	sciERC	7,169.87	$\clubsuit$	conll	sciERC	3,051.14	$\clubsuit$
conll	AnEM	7,186.44	$\clubsuit$	BTC	sec	4,274.04	$\clubsuit$
cerec	i2b2	7,927.04	$\clubsuit$	cerec	sciERC	4,769.50	$\clubsuit$

$\clubsuit$ indicates there is a shift detected. The full results of the full-sized dataset is in Table 10 and of the sampled dataset is in Table 11

#### MMD testing for input distribution

For the distributional representations, we apply MMD with a different number of samples (i.e. embedded sentences) from n = [5, 50, 200, 500, 1000, 2000]. Table 3 shows the results of these tests. The table is ordered by the scores generated using 2000 samples. We measure the difference between a dataset and itself as a sanity check.

Intuitively, the MMD test evaluates whether there is a significant difference between two distributions: a higher MMD value suggests a greater disparity between the distributions. The test essentially disproves the null hypothesis that the distributions are identical—when the MMD statistic is significantly high. In our study, the MMD values can appear negative due to estimation errors in smaller samples or due to the kernel choice affecting the calculation. However, the absolute value of MMD should be considered. Typically, a threshold for significance is set, above which the null hypothesis can be rejected. We use 0.05 as our threshold.

CoNLL, a widely used benchmark dataset in NER, is surprisingly far from other datasets in the distance measured by the chi-squared test. However, with MMD tests, the distance is fairly close. This is an indication that sentence-level representation provides more information than word-frequency representation.

#### Label distribution

To detect category shift in label distribution, we utilized the Chi-squared test, as detailed in subsection 3.4. This testing was performed on both the sampled and full-sized datasets, and the results are presented in Table 5. Results reveal a significant difference between the datasets that share the same categories and those that have different categories.

Datasets that are focused on specialized fields typically contain more specific labels. Consequently, the dissimilarity between these datasets and those from general domains is greater. For example, while the input shift between BTC and WNUT17 may be small, the label shift is relatively significant due to their distinct label spaces. For the NER task, generalizing model performance to datasets that have distinct categories is more challenging, as evidenced in the following section.

**Table 5:** Chi-squared testing for **label distributions** of full-sized datasets (left) and sampled datasets (948 samples) (right)

Source data	Target data	Statistics	Shift decision	Source data	Target data	Statistics	Shift decision
conll	conll	0.00		conll	conll	0.00	
cerec	cerec	0.00		cerec	cerec	0.00	
conll	wikigold	0.04		conll	wikigold	0.04	
BTC	sec	0.07		BTC	sec	0.10	
BTC	wikigold	0.51		BTC	wikigold	0.52	
BTC	WNUT17	0.84		BTC	WNUT17	0.84	
BTC	re3d	1.22		BTC	re3d	1.11	
.	.	.	.	.	.	.	.
conll	sciERC	311,849,153.00	$\clubsuit$	conll	sciERC	308,299,988.60	$\clubsuit$
i2b2-06	sciERC	320,519,601.10	$\clubsuit$	i2b2-06	sciERC	315,852,394.93	$\clubsuit$
BTC	sciERC	459,742,109.25	$\clubsuit$	BTC	sciERC	451,014,301.26	$\clubsuit$
sec	sciERC	555,622,639.19	$\clubsuit$	sec	sciERC	546,583,192.69	$\clubsuit$
cerec	sciERC	699,845,821.45	$\clubsuit$	cerec	sciERC	675,866,015.85	$\clubsuit$

The full results of the full-sized dataset is in Table 12 and of the sampled dataset is in Table 13

**Table 6:** Micro-average F1 score when the model is fine-tuned on the source dataset (the row) and tested on the target dataset (the column)

	conll	wikigold	cerec	BTC	re3d	i2b2-06	SEC	ontonotes	GUM	WNUT17	sciERC	AnEM	average f1
conll	**0**.**61**	0.38	0.24	0.28	0.21	0.13	0.13	0.23	0.06	0.24	0.00	0.00	**0**.**21**
wikigold	0.50	**0**.**51**	0.23	0.22	0.24	0.15	0.16	0.23	0.10	0.14	0.00	0.00	0.21
cerec	0.29	0.21	**0**.**74**	0.12	0.11	0.16	0.11	0.09	0.17	0.15	0.00	0.00	0.18
BTC	0.29	0.16	0.19	**0**.**62**	0.14	0.14	0.08	0.11	0.05	0.16	0.00	0.00	0.16
re3d	0.16	0.18	0.12	0.13	**0**.**47**	0.03	0.07	0.11	0.10	0.11	0.00	0.00	0.12
i2b2-06	0.09	0.09	0.12	0.17	0.01	**0**.**73**	0.02	0.02	0.01	0.07	0.00	0.00	0.11
SEC	0.09	0.05	0.06	0.06	0.02	0.00	**0**.**85**	0.08	0.01	0.05	0.00	0.00	0.11
ontonotes	0.14	0.08	0.03	0.05	0.11	0.02	0.04	**0**.**31**	0.03	0.05	0.00	0.00	0.07
GUM	0.08	0.09	0.12	0.05	0.08	0.01	0.03	0.03	**0**.**24**	0.04	0.00	0.00	0.06
WNUT17	0.08	0.07	0.11	0.07	0.02	0.08	0.01	0.02	0.02	**0**.**15**	0.00	0.00	0.05
sciERC	0.00	0.00	0.00	0.00	0.00	0.00	0.00	0.00	0.00	0.00	**0**.**24**	0.00	0.02
AnEM	0.00	0.00	0.00	0.00	0.00	0.00	0.00	0.00	0.00	0.00	0.00	**0**.**21**	0.02

Fine-tuning uses the BERT-base-uncased model. All performances are averaged over five trials. All datasets are sampled with 948 samples

**Table 7:** F1 scores on full-sized datasets

	conll	cerec	ontonotes	i2b2-06	wikigold	WNUT17	GUM	re3d	SEC	BTC	sciERC	AnEM	average f1
conll	**0**.**90**	0.35	0.31	0.13	0.62	0.26	0.12	0.33	0.10	0.31	0.00	0.00	**0**.**29**
cerec	0.35	**0**.**90**	0.13	0.10	0.31	0.14	0.21	0.19	0.08	0.20	0.00	0.00	0.25
ontonotes	0.28	0.14	**0**.**93**	0.05	0.26	0.14	0.08	0.17	0.06	0.17	0.00	0.00	0.23
i2b2-06	0.27	0.20	0.12	**0**.**99**	0.33	0.06	0.05	0.22	0.02	0.16	0.00	0.00	0.22
wikigold	0.57	0.25	0.30	0.10	**0**.**88**	0.13	0.13	0.30	0.13	0.20	0.00	0.00	0.21
WNUT17	0.43	0.32	0.29	0.15	0.44	**0**.**70**	0.10	0.24	0.09	0.27	0.00	0.00	0.21
GUM	0.20	0.17	0.05	0.01	0.23	0.04	**0**.**68**	0.28	0.04	0.06	0.00	0.00	0.21
re3d	0.19	0.25	0.15	0.04	0.19	0.11	0.14	**0**.**61**	0.06	0.20	0.00	0.00	0.21
SEC	0.22	0.14	0.19	0.05	0.25	0.09	0.05	0.12	**0**.**93**	0.21	0.00	0.00	0.21
BTC	0.59	0.35	0.32	0.14	0.62	0.24	0.14	0.34	0.15	**0**.**87**	0.00	0.00	0.20
sciERC	0.00	0.00	0.00	0.00	0.00	0.00	0.00	0.00	0.00	0.00	**0**.**57**	0.00	0.19
AnEM	0.00	0.00	0.00	0.00	0.00	0.00	0.00	0.00	0.00	0.00	0.00	**0**.**79**	0.19

Fine-tuning uses the BERT-base model

**Table 8:** F1 scores on full-sized datasets

	conll	cerec	ontonotes	i2b2-06	wikigold	WNUT17	GUM	re3d	SEC	BTC	sciERC	AnEM	average f1
conll	**0**.**88**	0.32	0.26	0.11	0.58	0.21	0.10	0.29	0.36	0.23	0.00	0.00	**0**.**28**
cerec	0.30	**0**.**87**	0.11	0.06	0.30	0.12	0.20	0.20	0.31	0.15	0.00	0.00	0.25
ontonotes	0.23	0.14	**0**.**92**	0.10	0.22	0.12	0.07	0.15	0.07	0.15	0.00	0.00	0.23
i2b2-06	0.33	0.22	0.17	**1**.**00**	0.36	0.09	0.09	0.26	0.23	0.16	0.00	0.00	0.23
wikigold	0.49	0.25	0.29	0.10	**0**.**84**	0.11	0.11	0.28	0.38	0.15	0.00	0.00	0.21
WNUT17	0.40	0.24	0.25	0.14	0.43	**0**.**61**	0.09	0.23	0.19	0.25	0.00	0.00	0.21
GUM	0.15	0.16	0.04	0.03	0.21	0.03	**0**.**63**	0.26	0.04	0.06	0.00	0.00	0.21
re3d	0.15	0.21	0.11	0.03	0.16	0.07	0.13	**0**.**53**	0.09	0.11	0.00	0.00	0.20
SEC	0.21	0.19	0.15	0.11	0.18	0.08	0.05	0.10	**0**.**90**	0.21	0.00	0.00	0.20
BTC	0.50	0.33	0.32	0.14	0.58	0.22	0.12	0.30	0.31	**0**.**85**	0.00	0.00	0.20
sciERC	0.00	0.00	0.00	0.00	0.00	0.00	0.00	0.00	0.00	0.00	**0**.**55**	0.00	0.19
AnEM	0.00	0.00	0.00	0.00	0.00	0.00	0.00	0.00	0.00	0.00	0.00	**0**.**77**	0.19

Fine-tuning uses the BioBERT-base model

**Fig. 7:**
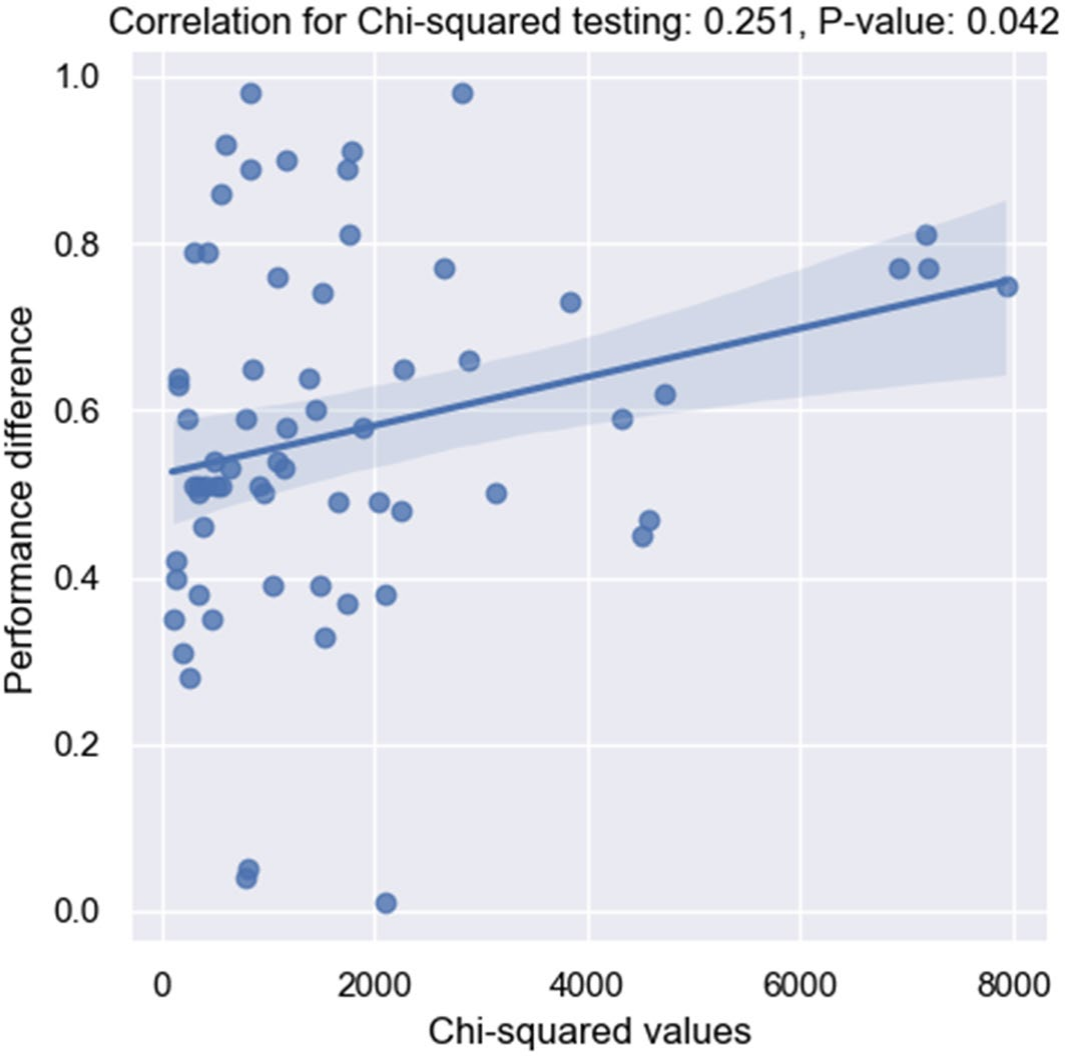
Plots for Chi-squared measures with word frequency input distribution and performance difference. Linear regression model fitted

**Fig. 8:**
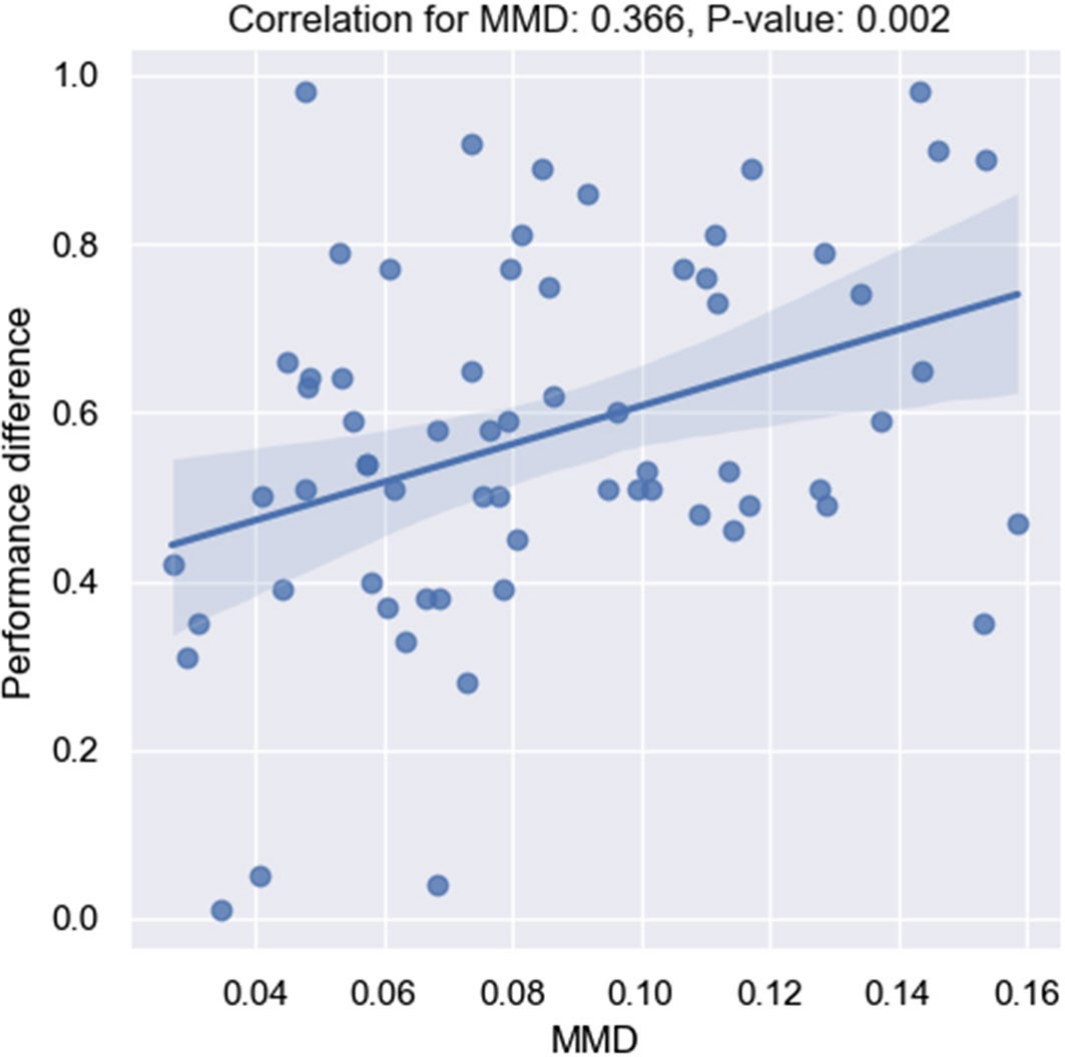
Plots for MMD measures with sentence-level input distributions and performance difference. Linear regression model fitted

**Fig. 9:**
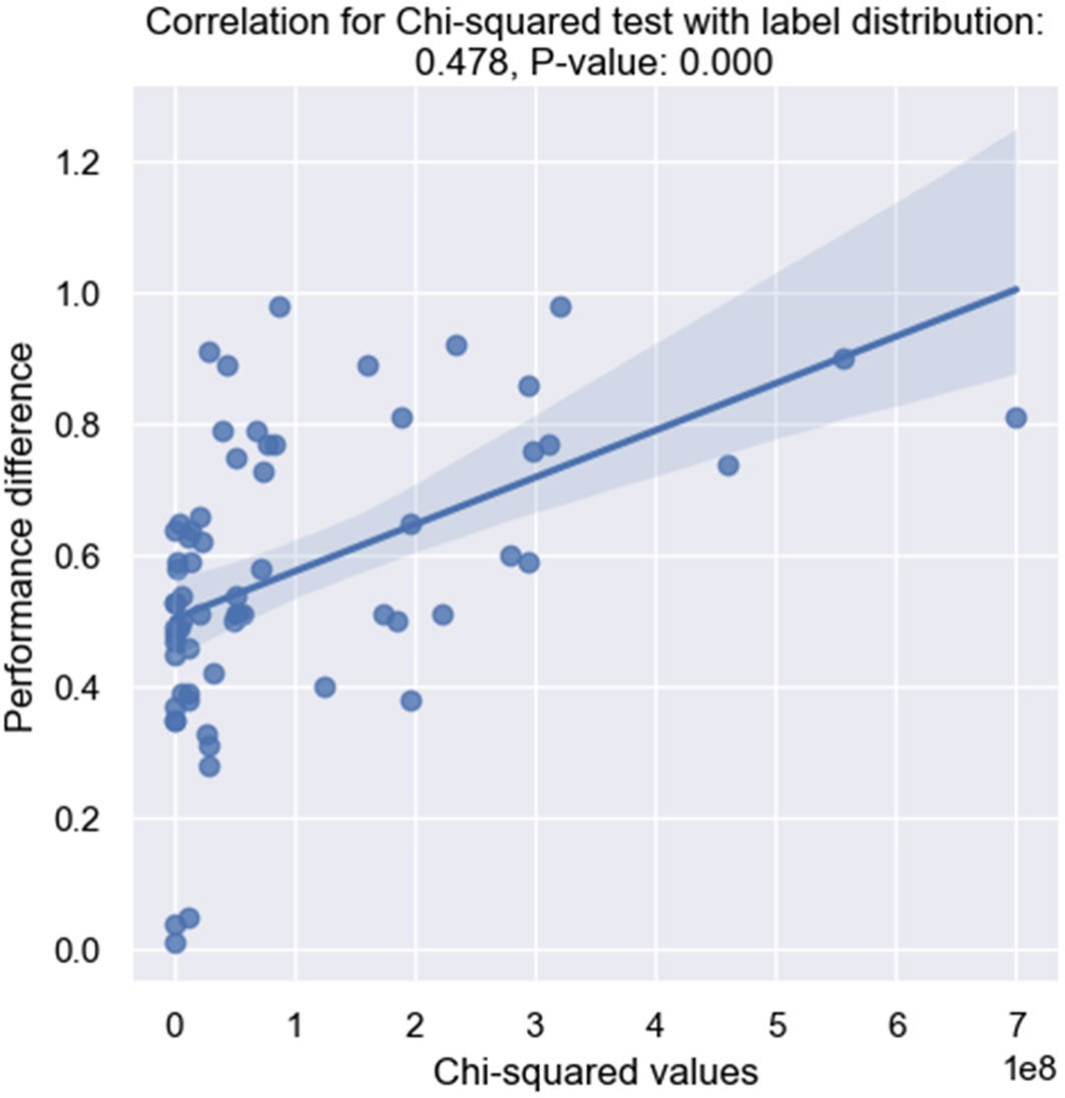
Plots for Chi-squared measures with label distribution and performance difference. Linear regression model fitted

### Performance measurement

As noted in subsection 3.4, we conducted four sets of experiments, including fine-tuning models on both original-sized and sampled datasets with 948 samples using both BERT-base and BioBERT-base models. All performance results are reported using the average of five trials with different random sampling. The complete results can be seen in Appendix B.

Tables 6, 7, 8 present the micro-averaged F1 performance of the models trained and tested on the sampled datasets with BERT, full-sized datasets with BERT, and full-sized datasets with BioBERT, respectively. Each row in the table indicates the dataset the model is fine-tuned on. Correspondingly, the columns indicate the dataset which the fine-tuned model is tested upon. All performance results reported here are the average of 5 trials. The standard deviation of these results ranges from 0.0 to 0.07. The last column reports the average F1 scores of all the test results, which represents the generalization ability of the model when fine-tuned on a specific dataset. The rows are ordered by this average F1 score.

Table 7 reveals that even though BTC and WNUT17 contain texts from the same domain, the model’s generalization ability on WNUT17 decreases significantly when fine-tuned on BTC, as there is a significant label category shift between these two datasets.

Comparing Tables 6 and Table 7, we observe that when we control the number of data samples, the average F1 scores tend to decrease. However, the overall rankings of the datasets are similar between the two tables, except for WNUT17 and Re3d. Using a subset of the original dataset reduces the generalization ability significantly, indicating the additional data samples in the original data set help improve the generalization. Conversely, for dataset Re3d, the generalization ability increases while the number of samples decreases, indicating that the additional data samples in the dataset harm the generalization.

Due to mutually exclusive sets of categories, we encounter many zero F1 scores on AnEM dataset and SciERC dataset. Even though fine-tuning helps improve the performance on the same dataset, the generalization ability is low, indicating that category shift has a significant impact on the performance.

Comparing Tables 7 and 8, we observe that the fine-tuning performance is slightly impacted by the text on which these language models were pre-trained. However, the average F1 score rankings remain the same.

### Correlation

To investigate further how shifts impact model performance, we report the correlation between the testing statistics and performance differences in Figs. 7, 8, 9. Within each plot, the x-axis represents the distances calculated by different hypothesis testing, and the y-axis represents the performance differences between a source dataset and a target dataset. The higher the performance distance is, the worse the generalization ability the model exhibits. Assuming there are datasets $D_a$ and $D_b$. $Perf_{ab}$ indicates the performance difference on $D_a$ and $D_b$ when the model is fine-tuned on source dataset $D_s$ where $s \in \{D_i \mid i=\{1,2,\ldots ,12\}\}$. $Shift_{ab}$ is the distance between $D_a$ and $D_b$ with regarding to each statistical test. The correlation is calculated between *perf* and *shift*.

Based on the presented plots, it is evident that the label category shift shows the most statistically significant correlation (P <0.0001) with model performance. This finding suggests that category shift can serve as a reliable indicator of model performance in a supervised setting when evaluating in a new domain. With respect to input distribution shift, while the word frequency distribution’s correlation with model performance is the lowest, it is still significant (P =0.042). The MMD results reveal a moderately strong correlation (P = 0.002). This indicates that in an unsupervised setting, MMD testing with sentence-level representation distribution can be used to estimate model performance when transferring between domains.

## Conclusion

In this work, we investigated input data and label distribution shifts across 12 benchmark NER datasets. We compared two different types of representations for input shifts. We systematically measured the shifts using the lens of statistical testing. We measured performance differences by fine-tuning BERT models and calculating the correlation between shifts and performance.

The results show that both word frequency distribution and sentence-level distributional representations are useful for ascertaining shift. Changing between domains results in measurable differences in distribution shifts. Results show that label shift correlates more significantly with performance degradation than input shifts for NER. However, there is still a correlation between input shift and performance degradation. Here, sentence-level representations provide more signals for the relation between distribution shift and performance.

Based on these results, we believe that shift detection and the measurement of distribution shifts can play important roles in tackling NLP tasks, especially for new and low-resource domains. In particular, when applying a model to a new domain, or as data changes, the measures detailed above can help researchers and practitioners decide whether the expense of gathering new annotated data and subsequent fine-tuning is warranted. In the future, we hope that distribution shift measurement can become part of widely used NLP paradigms such as crowd-sourcing and active learning.^4^

## Figures and Tables

**Fig. 10:**
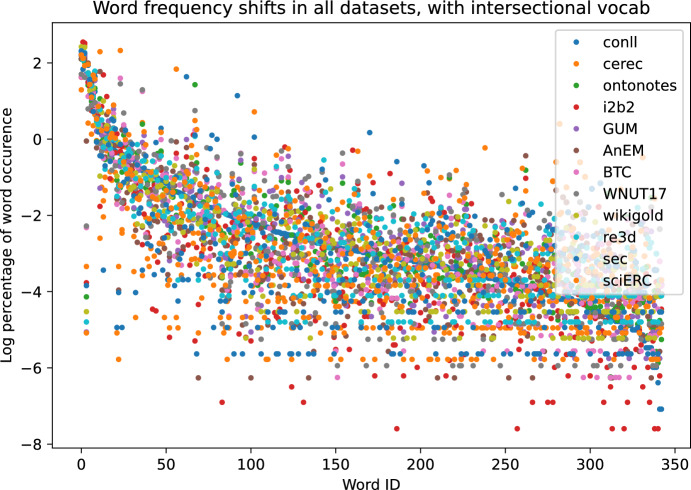
Frequency plots across all datasets with an intersectional vocabulary where the vocabulary is an intersection of all vocabularies

## Appendix A: Label mapping

We provide the dictionary of type mapping across datasets in the following.

## Appendix B: Full results

See Tables 9, 10, 11, 12, 13, 14, 15, 16 and Fig. 10.

**Table 9:** MMD statistics for all combination without repetition of datasets with different number of samples

Source data	Target data	Number of samples from test					
		5	50	200	500	948	2,000
conll	conll	− 0.30	− 0.03	− 0.01	− 0.0029	− 0.0015	− 0.0007
cerec	cerec	− 0.29	− 0.03	− 0.01	− 0.0029	− 0.0015	− 0.0007
ontonotes	ontonotes	− 0.30	− 0.03	− 0.01	− 0.0029	− 0.0015	− 0.0007
i2b2-06	i2b2-06	− 0.29	− 0.03	− 0.01	− 0.0028	− 0.0015	− 0.0007
GUM	GUM	− 0.30	− 0.03	− 0.01	− 0.0030	− 0.0016	− 0.0007
AnEM	AnEM	− 0.30	− 0.03	− 0.01	− 0.0029	− 0.0015	− 0.0007
BTC	BTC	− 0.29	− 0.03	− 0.01	− 0.0029	− − 0.0015	− 0.0007
WNUT17	WNUT17	− 0.30	− 0.03	− 0.01	− 0.0029	− 0.0015	− 0.0007
wikigold	wikigold	− 0.30	− 0.03	− 0.01	− 0.0030	− 0.0016	− 0.0007
re3d	re3d	− 0.28	− 0.03	− 0.01	− 0.0028	− 0.0015	− 0.0007
SEC	SEC	− 0.29	− 0.03	− 0.01	− 0.0028	− 0.0015	− 0.0007
sciERC	sciERC	− 0.29	− 0.03	− 0.01	− 0.0029	− 0.0015	− 0.0007
BTC	WNUT17	0.02	0.01*	0.01*	0.01*	0.01*	0.01*
GUM	wikigold	0.00	0.02*	0.02*	0.02*	0.02*	0.02*
conll	wikigold	0.03*	0.04*	0.03*	0.03*	0.03*	0.03*
ontonotes	GUM	0.02	0.02*	0.04*	0.03*	0.03*	0.03*
GUM	WNUT17	0.03	0.04*	0.03*	0.03*	0.03*	0.03*
conll	GUM	0.02	0.03*	0.04*	0.03*	0.04*	0.04*
GUM	BTC	0.04*	0.04*	0.04*	0.04*	0.04*	0.04*
GUM	AnEM	0.04*	0.03*	0.04*	0.04*	0.04*	0.04*
ontonotes	wikigold	0.04*	0.04*	0.05*	0.04*	0.04*	0.04*
ontonotes	BTC	0.02	0.04*	0.04*	0.04*	0.04*	0.04*
conll	ontonotes	0.01	0.04*	0.04*	0.04*	0.04*	0.04*
AnEM	wikigold	0.08*	0.05*	0.05*	0.05*	0.05*	0.05*
ontonotes	WNUT17	0.05*	0.03*	0.04*	0.04*	0.05*	0.05*
WNUT17	wikigold	$-$0.01	0.04*	0.05*	0.05*	0.05*	0.05*
conll	WNUT17	0.12*	0.06*	0.04*	0.05*	0.05*	0.05*
conll	BTC	0.06	0.05*	0.05*	0.05*	0.05*	0.05*
i2b2-06	AnEM	0.04	0.04*	0.05*	0.05*	0.05*	0.05*
BTC	wikigold	0.09*	0.05*	0.05*	0.05*	0.05*	0.05*
cerec	WNUT17	0.08*	0.06*	0.06*	0.06*	0.06*	0.06*
ontonotes	re3d	0.02	0.05*	0.06*	0.06*	0.05*	0.06*
cerec	GUM	0.08*	0.06*	0.06*	0.06*	0.06*	0.06*
conll	AnEM	0.08*	0.06*	0.06*	0.06*	0.06*	0.06*
conll	cerec	0.14*	0.07*	0.05*	0.06*	0.06*	0.06*
cerec	BTC	0.06*	0.05*	0.06*	0.06*	0.06*	0.06*
GUM	re3d	0.05*	0.05*	0.07*	0.06*	0.06*	0.06*
cerec	wikigold	0.05*	0.07*	0.05*	0.07*	0.06*	0.06*
cerec	ontonotes	0.04	0.06*	0.06*	0.06*	0.07*	0.06*
ontonotes	AnEM	0.07*	0.07*	0.07*	0.06*	0.07*	0.07*
i2b2-06	wikigold	0.04	0.09*	0.07*	0.07*	0.07*	0.07*
conll	i2b2-06	0.05	0.07*	0.07*	0.07*	0.07*	0.07*
i2b2-06	GUM	0.07	0.07*	0.07*	0.07*	0.07*	0.07*
AnEM	WNUT17	0.04*	0.07*	0.08*	0.07*	0.07*	0.07*
GUM	sciERC	0.06*	0.08*	0.07*	0.07*	0.07*	0.07*
conll	re3d	0.09*	0.06*	0.07*	0.07*	0.07*	0.08*
wikigold	re3d	0.04	0.08*	0.08*	0.08*	0.08*	0.08*
AnEM	BTC	0.10*	0.08*	0.08*	0.08*	0.08*	0.08*
cerec	i2b2-06	0.04	0.09*	0.08*	0.09*	0.08*	0.08*
i2b2-06	WNUT17	0.07*	0.10*	0.09*	0.09*	0.09*	0.08*
cerec	AnEM	0.02	0.08*	0.09*	0.08*	0.08*	0.09*
BTC	re3d	0.08*	0.10*	0.08*	0.08*	0.08*	0.09*
wikigold	sciERC	0.05*	0.10*	0.09*	0.09*	0.09*	0.09*
i2b2-06	BTC	0.05*	0.10*	0.10*	0.09*	0.09*	0.09*
WNUT17	re3d	0.12*	0.11*	0.08*	0.09*	0.09*	0.09*
ontonotes	sciERC	0.12*	0.09*	0.10*	0.09*	0.09*	0.09*
ontonotes	i2b2-06	0.12*	0.08*	0.10*	0.10*	0.10*	0.10*
AnEM	sciERC	0.14*	0.09*	0.10*	0.09*	0.10*	0.10*
conll	sciERC	0.09*	0.11*	0.11*	0.10*	0.10*	0.10*
AnEM	re3d	0.08*	0.10*	0.11*	0.10*	0.10*	0.10*
WNUT17	sciERC	0.07*	0.09*	0.10*	0.10*	0.10*	0.10*
cerec	re3d	0.04*	0.11*	0.10*	0.10*	0.10*	0.10*
cerec	SEC	0.13*	0.12*	0.12*	0.11*	0.11*	0.11*
BTC	sciERC	0.08*	0.11*	0.11*	0.11*	0.11*	0.11*
cerec	sciERC	0.15*	0.11*	0.11*	0.11*	0.11*	0.11*
conll	SEC	0.13*	0.13*	0.12*	0.11*	0.12*	0.11*
GUM	SEC	0.11*	0.10*	0.11*	0.11*	0.11*	0.11*
wikigold	SEC	0.19*	0.09*	0.12*	0.12*	0.12*	0.11*
ontonotes	SEC	0.02	0.11*	0.11*	0.11*	0.11*	0.12*
AnEM	SEC	0.10*	0.14*	0.13*	0.13*	0.12*	0.13*
i2b2-06	re3d	0.13*	0.11*	0.13*	0.12*	0.13*	0.13*
re3d	sciERC	0.17*	0.13*	0.13*	0.13*	0.14*	0.14*
i2b2-06	sciERC	0.12*	0.15*	0.14*	0.14*	0.14*	0.14*
BTC	SEC	0.10*	0.13*	0.15*	0.15*	0.14*	0.15*
WNUT17	SEC	0.16*	0.14*	0.14*	0.14*	0.14*	0.15*
i2b2-06	SEC	0.17*	0.16*	0.16*	0.15*	0.15*	0.15*
SEC	sciERC	0.11*	0.13*	0.15*	0.16*	0.15*	0.15*
re3d	SEC	0.20*	0.15*	0.15*	0.15*	0.16*	0.15*

The table is ordered by the distance between pairs of datasets when the number of samples are 2000. All results are averaged over five trials. The star signs show the results are statistically significant (*p* value <.05) and there are shifts detected. This the full results of the Table 3

**Table 10:** Input Chi-squared statistics for all combinations without repetition of datasets

Source data	Target data	Statistics	Shift decision
conll	conll	0.00	
cerec	cerec	0.00	
ontonotes	ontonotes	0.00	
i2b2	i2b2	0.00	
GUM	GUM	0.00	
AnEM	AnEM	0.00	
BTC	BTC	0.00	
WNUT17	WNUT17	0.00	
wikigold	wikigold	0.00	
re3d	re3d	0.00	
sec	sec	0.00	
sciERC	sciERC	0.00	
BTC	WNUT17	96.04	
GUM	WNUT17	123.53	
GUM	BTC	127.48	
ontonotes	WNUT17	144.48	
ontonotes	re3d	149.69	
GUM	wikigold	191.42	
wikigold	re3d	232.63	
GUM	re3d	257.93	
ontonotes	GUM	292.86	
AnEM	re3d	302.56	
AnEM	wikigold	337.45	
ontonotes	wikigold	346.67	
GUM	sciERC	349.31	
GUM	sec	385.12	
AnEM	WNUT17	401.39	
i2b2	re3d	426.14	
re3d	sec	471.70	
ontonotes	BTC	479.08	
AnEM	BTC	500.50	
wikigold	sciERC	544.95	
AnEM	sec	548.65	
i2b2	GUM	584.29	
wikigold	sec	642.16	
BTC	wikigold	787.59	
re3d	sciERC	789.35	
WNUT17	wikigold	794.37	
ontonotes	AnEM	820.95	
i2b2	AnEM	823.57	
ontonotes	sec	853.52	
AnEM	sciERC	913.34	
GUM	AnEM	961.50	
conll	WNUT17	1,047.27	
i2b2	BTC	1,086.96	
conll	cerec	1,089.80	
cerec	re3d	1,148.76	
i2b2	wikigold	1,164.45	
sec	sciERC	1,171.78	
cerec	WNUT17	1,378.34	
WNUT17	sciERC	1,434.51	
WNUT17	re3d	1,478.79	
BTC	sciERC	1,510.25	
conll	BTC	1,524.60	
WNUT17	sec	1,656.90	
cerec	wikigold	1,727.73	$\clubsuit$
ontonotes	sciERC	1,728.98	
cerec	AnEM	1,757.95	$\clubsuit$
i2b2	sec	1,777.99	
cerec	BTC	1,879.36	
conll	sec	2,027.94	
conll	ontonotes	2,092.56	
conll	wikigold	2,100.30	
cerec	sec	2,240.83	$\clubsuit$
conll	i2b2	2,257.31	
i2b2	WNUT17	2,653.34	
i2b2	sciERC	2,808.05	$\clubsuit$
conll	GUM	2,877.06	
conll	re3d	3,124.31	$\clubsuit$
ontonotes	i2b2	3,838.24	
cerec	GUM	4,310.95	$\clubsuit$
BTC	re3d	4,513.72	$\clubsuit$
BTC	sec	4,568.51	$\clubsuit$
cerec	ontonotes	4,726.86	$\clubsuit$
conll	sciERC	6,930.89	$\clubsuit$
cerec	sciERC	7,169.87	$\clubsuit$
conll	AnEM	7,186.44	$\clubsuit$
cerec	i2b2	7,927.04	$\clubsuit$

The table is ordered by the chi-squared value following ascending order. All word occurrences that below 5 are filtered for the effective usage of Chi-suared testing. The symbol $\clubsuit$ indicates there are shifts detected. This is the full results of the left table in Table 4

**Table 11:** Input Chi-squared statistics for sampled datasets

Source data	Target data	Statistics	Shift decision
conll	conll	0.00	
cerec	cerec	0.00	
ontonotes	ontonotes	0.00	
i2b2	i2b2	0.00	
GUM	GUM	0.00	
AnEM	AnEM	0.00	
BTC	BTC	0.00	
WNUT17	WNUT17	0.00	
wikigold	wikigold	0.00	
re3d	re3d	0.00	
sec	sec	0.00	
sciERC	sciERC	0.00	
GUM	BTC	75.67	
GUM	WNUT17	81.64	
BTC	WNUT17	93.89	
ontonotes	WNUT17	98.60	
ontonotes	BTC	124.59	
ontonotes	re3d	131.39	
AnEM	BTC	158.86	
GUM	wikigold	162.41	
i2b2	wikigold	172.97	
AnEM	WNUT17	189.54	
wikigold	sciERC	191.47	
AnEM	re3d	195.15	
GUM	re3d	207.65	
i2b2	GUM	211.51	
wikigold	re3d	213.81	
GUM	AnEM	214.20	
GUM	sciERC	244.28	
ontonotes	wikigold	249.68	
i2b2	AnEM	258.37	
conll	AnEM	258.89	
ontonotes	AnEM	283.88	
AnEM	wikigold	292.75	
conll	BTC	295.26	
i2b2	BTC	296.28	
re3d	sec	316.50	
i2b2	re3d	320.14	
wikigold	sec	327.45	
ontonotes	GUM	345.13	
ontonotes	i2b2	357.75	
GUM	sec	401.75	
re3d	sciERC	404.84	
WNUT17	re3d	407.13	
AnEM	sec	433.05	
conll	re3d	433.08	
i2b2	WNUT17	490.46	
sec	sciERC	501.09	
cerec	WNUT17	603.98	
AnEM	sciERC	635.92	
ontonotes	sec	716.12	
BTC	wikigold	748.72	
conll	sec	760.04	
conll	WNUT17	778.92	
cerec	re3d	815.43	$\clubsuit$
conll	GUM	832.89	
cerec	wikigold	836.13	$\clubsuit$
WNUT17	wikigold	861.27	
cerec	i2b2	875.33	$\clubsuit$
i2b2	sec	881.32	$\clubsuit$
cerec	AnEM	892.46	$\clubsuit$
i2b2	sciERC	905.32	$\clubsuit$
cerec	GUM	1,89.98	$\clubsuit$
conll	i2b2	1,425.79	$\clubsuit$
cerec	sec	1,428.67	$\clubsuit$
BTC	sciERC	1,643.86	$\clubsuit$
cerec	BTC	1,795.81	$\clubsuit$
conll	cerec	1,810.52	$\clubsuit$
cerec	ontonotes	1,961.29	$\clubsuit$
WNUT17	sciERC	2,16.53	$\clubsuit$
conll	wikigold	2,145.54	$\clubsuit$
conll	ontonotes	2,348.69	$\clubsuit$
BTC	re3d	2,480.40	$\clubsuit$
WNUT17	sec	2,590.02	$\clubsuit$
ontonotes	sciERC	2,608.52	$\clubsuit$
conll	sciERC	3,51.15	$\clubsuit$
BTC	sec	4,274.04	$\clubsuit$
cerec	sciERC	4,769.50	$\clubsuit$

The symbol $\clubsuit$ indicates there are shifts detected. This is the full results of the right table in Table 4

**Table 12:** Label distribution Chi-squared testing statistics for all combinations without repetition of datasets

Source data	Target data	Statistics	Shift Decision
conll	conll	0.00	
cerec	cerec	0.00	
ontonotes	ontonotes	0.00	
i2b2-06	i2b2-06	0.00	
GUM	GUM	0.00	
AnEM	AnEM	0.00	
BTC	BTC	0.00	
WNUT17	WNUT17	0.00	
wikigold	wikigold	0.00	
re3d	re3d	0.00	
sec	sec	0.00	
sciERC	sciERC	0.00	
conll	wikigold	0.04	
BTC	sec	0.07	
BTC	wikigold	0.51	
BTC	WNUT17	0.84	
BTC	re3d	1.22	
conll	sec	2.03	
wikigold	sec	4.24	
cerec	sec	326,074.25	$\clubsuit$
cerec	re3d	745,315.20	$\clubsuit$
cerec	wikigold	781,788.25	$\clubsuit$
cerec	WNUT17	841,992.77	$\clubsuit$
re3d	sec	1,310,152.93	$\clubsuit$
cerec	GUM	2,564,429.92	$\clubsuit$
cerec	BTC	3,257,665.19	$\clubsuit$
WNUT17	sec	5,112,738.62	$\clubsuit$
ontonotes	sec	5,223,820.29	$\clubsuit$
conll	cerec	5,780,109.23	$\clubsuit$
conll	re3d	6,382,202.06	$\clubsuit$
conll	WNUT17	7,210,082.54	$\clubsuit$
WNUT17	re3d	11,686,258.40	$\clubsuit$
ontonotes	re3d	11,940,158.70	$\clubsuit$
WNUT17	wikigold	12,258,168.10	$\clubsuit$
GUM	sec	12,466,422.81	$\clubsuit$
ontonotes	wikigold	12,524,494.36	$\clubsuit$
ontonotes	WNUT17	13,489,000.18	$\clubsuit$
wikigold	re3d	13,583,255.59	$\clubsuit$
conll	GUM	21,959,515.63	$\clubsuit$
AnEM	sec	22,315,222.59	$\clubsuit$
cerec	ontonotes	22,872,211.25	$\clubsuit$
conll	BTC	27,895,788.79	$\clubsuit$
GUM	re3d	28,494,675.69	$\clubsuit$
i2b2-06	sec	29,859,890.76	$\clubsuit$
GUM	wikigold	29,889,167.66	$\clubsuit$
GUM	WNUT17	32,190,918.71	$\clubsuit$
ontonotes	GUM	41,083,014.26	$\clubsuit$
ontonotes	AnEM	43,451,712.45	$\clubsuit$
GUM	AnEM	50,349,808.09	$\clubsuit$
AnEM	re3d	51,006,213.20	$\clubsuit$
cerec	i2b2-06	51,746,456.14	$\clubsuit$
ontonotes	BTC	52,188,909.35	$\clubsuit$
AnEM	wikigold	53,502,390.51	$\clubsuit$
AnEM	WNUT17	57,622,585.01	$\clubsuit$
i2b2-06	re3d	68,251,165.27	$\clubsuit$
i2b2-06	wikigold	71,591,287.74	$\clubsuit$
ontonotes	i2b2-06	74,740,292.02	$\clubsuit$
i2b2-06	WNUT17	77,104,499.80	$\clubsuit$
conll	AnEM	84,469,257.53	$\clubsuit$
i2b2-06	AnEM	86,817,785.05	$\clubsuit$
GUM	BTC	124,546,587.29	$\clubsuit$
ontonotes	sciERC	160,417,887.32	$\clubsuit$
AnEM	sciERC	174,040,643.86	$\clubsuit$
GUM	sciERC	185,884,729.65	$\clubsuit$
cerec	AnEM	189,564,270.53	$\clubsuit$
conll	ontonotes	195,857,558.82	$\clubsuit$
conll	i2b2-06	197,142,187.31	$\clubsuit$
AnEM	BTC	222,941,642.42	$\clubsuit$
i2b2-06	GUM	234,834,691.92	$\clubsuit$
WNUT17	sciERC	279,748,960.06	$\clubsuit$
wikigold	sciERC	293,835,211.87	$\clubsuit$
re3d	sciERC	294,915,993.80	$\clubsuit$
i2b2-06	BTC	298,317,122.09	$\clubsuit$
conll	sciERC	311,849,153.00	$\clubsuit$
i2b2-06	sciERC	320,519,601.10	$\clubsuit$
BTC	sciERC	459,742,109.25	$\clubsuit$
sec	sciERC	555,622,639.19	$\clubsuit$

The table is ordered by the test value in ascending order. This is the full results of the left table in Table 5

**Table 13:** Label distribution Chi-squared testing statistics for sampled datasets

Source data	Target data	Statistics	Shift Decision
conll	conll	0.00	
cerec	cerec	0.00	
ontonotes	ontonotes	0.00	
i2b2-06	i2b2-06	0.00	
GUM	GUM	0.00	
AnEM	AnEM	0.00	
BTC	BTC	0.00	
WNUT17	WNUT17	0.00	
wikigold	wikigold	0.00	
re3d	re3d	0.00	
sec	sec	0.00	
sciERC	sciERC	0.00	
conll	wikigold	0.04	
BTC	sec	0.10	
BTC	wikigold	0.52	
BTC	WNUT17	0.84	
BTC	re3d	1.11	
conll	sec	1.47	
wikigold	sec	2.60	
cerec	sec	334,179.62	$\clubsuit$
cerec	wikigold	839,683.36	$\clubsuit$
cerec	re3d	843,668.88	$\clubsuit$
cerec	WNUT17	917,624.79	$\clubsuit$
re3d	sec	1,066,254.19	$\clubsuit$
cerec	GUM	2,900,540.31	$\clubsuit$
cerec	BTC	3,687,180.00	$\clubsuit$
WNUT17	sec	4,377,314.31	$\clubsuit$
ontonotes	sec	4,780,033.07	$\clubsuit$
conll	cerec	5,836,613.50	$\clubsuit$
conll	re3d	6,491,937.00	$\clubsuit$
conll	WNUT17	7,061,035.89	$\clubsuit$
WNUT17	wikigold	10,998,734.44	$\clubsuit$
WNUT17	re3d	11,050,923.20	$\clubsuit$
GUM	sec	11,530,780.73	$\clubsuit$
wikigold	re3d	11,602,120.49	$\clubsuit$
ontonotes	wikigold	12,010,632.37	$\clubsuit$
ontonotes	re3d	12,067,622.20	$\clubsuit$
ontonotes	WNUT17	13,125,498.34	$\clubsuit$
AnEM	sec	20,244,050.26	$\clubsuit$
conll	GUM	22,319,385.77	$\clubsuit$
cerec	ontonotes	26,018,437.52	$\clubsuit$
i2b2-06	sec	26,465,192.08	$\clubsuit$
conll	BTC	28,372,520.38	$\clubsuit$
GUM	wikigold	28,973,014.22	$\clubsuit$
GUM	re3d	29,110,489.58	$\clubsuit$
GUM	WNUT17	31,662,383.01	$\clubsuit$
ontonotes	GUM	41,488,682.25	$\clubsuit$
ontonotes	AnEM	44,092,822.88	$\clubsuit$
AnEM	wikigold	50,866,559.82	$\clubsuit$
GUM	AnEM	50,979,958.03	$\clubsuit$
AnEM	re3d	51,107,918.60	$\clubsuit$
ontonotes	BTC	52,740,634.40	$\clubsuit$
AnEM	WNUT17	55,588,158.29	$\clubsuit$
cerec	i2b2-06	59,943,853.08	$\clubsuit$
i2b2-06	wikigold	66,498,217.78	$\clubsuit$
i2b2-06	re3d	66,813,748.00	$\clubsuit$
i2b2-06	WNUT17	72,670,797.12	$\clubsuit$
ontonotes	i2b2-06	74,879,460.31	$\clubsuit$
conll	AnEM	85,589,611.44	$\clubsuit$
i2b2-06	AnEM	87,686,295.03	$\clubsuit$
GUM	BTC	127,225,200.96	$\clubsuit$
ontonotes	sciERC	158,825,547.52	$\clubsuit$
AnEM	sciERC	173,009,643.23	$\clubsuit$
GUM	sciERC	183,633,507.68	$\clubsuit$
cerec	AnEM	187,632,540.01	$\clubsuit$
conll	i2b2-06	195,573,212.31	$\clubsuit$
conll	ontonotes	200,209,544.04	$\clubsuit$
AnEM	BTC	223,363,307.19	$\clubsuit$
i2b2-06	GUM	229,706,748.40	$\clubsuit$
WNUT17	sciERC	279,425,297.10	$\clubsuit$
i2b2-06	BTC	292,004,448.24	$\clubsuit$
wikigold	sciERC	294,289,564.04	$\clubsuit$
re3d	sciERC	305,681,552.91	$\clubsuit$
conll	sciERC	308,299,988.60	$\clubsuit$
i2b2-06	sciERC	315,852,394.93	$\clubsuit$
BTC	sciERC	451,014,301.26	$\clubsuit$
sec	sciERC	546,583,192.69	$\clubsuit$

This is the full result of the right table in Table 5

**Table 14:** F1 scores on full-sized datasets

	conll	cerec	ontonotes	i2b2-06	wikigold	WNUT17	GUM	re3d	SEC	BTC	sciERC	AnEM	average f1
conll	**0**.**90**	0.35	0.31	0.13	0.62	0.26	0.12	0.33	0.10	0.31	0.00	0.00	**0**.**29**
cerec	0.35	**0**.**90**	0.13	0.10	0.31	0.14	0.21	0.19	0.08	0.20	0.00	0.00	0.25
ontonotes	0.28	0.14	**0**.**93**	0.05	0.26	0.14	0.08	0.17	0.06	0.17	0.00	0.00	0.23
i2b2-06	0.27	0.20	0.12	**0**.**99**	0.33	0.06	0.05	0.22	0.02	0.16	0.00	0.00	0.22
wikigold	0.57	0.25	0.30	0.10	**0**.**88**	0.13	0.13	0.30	0.13	0.20	0.00	0.00	0.21
WNUT17	0.43	0.32	0.29	0.15	0.44	**0**.**70**	0.10	0.24	0.09	0.27	0.00	0.00	0.21
GUM	0.20	0.17	0.05	0.01	0.23	0.04	**0**.**68**	0.28	0.04	0.06	0.00	0.00	0.21
re3d	0.19	0.25	0.15	0.04	0.19	0.11	0.14	**0**.**61**	0.06	0.20	0.00	0.00	0.21
SEC	0.22	0.14	0.19	0.05	0.25	0.09	0.05	0.12	**0**.**93**	0.21	0.00	0.00	0.21
BTC	0.59	0.35	0.32	0.14	0.62	0.24	0.14	0.34	0.15	**0**.**87**	0.00	0.00	0.20
sciERC	0.00	0.00	0.00	0.00	0.00	0.00	0.00	0.00	0.00	0.00	**0**.**57**	0.00	0.19
AnEM	0.00	0.00	0.00	0.00	0.00	0.00	0.00	0.00	0.00	0.00	0.00	**0**.**79**	0.19

Fine-tuning uses the BERT-base model

**Table 15:** Average F1 scores of 5 trials on sampled datasets (948 samples)

	conll	wikigold	BTC	cerec	re3d	i2b2-06	ontonotes	SEC	WNUT17	GUM	sciERC	AnEM	average f1
conll	**0**.**61**	0.40	0.28	0.24	0.18	0.17	0.24	0.12	0.25	0.07	0.00	0.00	**0**.**21**
wikigold	0.46	**0**.**54**	0.13	0.15	0.25	0.15	0.21	0.14	0.11	0.10	0.00	0.00	0.19
BTC	0.35	0.26	**0**.**64**	0.21	0.17	0.16	0.14	0.09	0.17	0.06	0.00	0.00	0.19
cerec	0.23	0.18	0.11	**0**.**70**	0.10	0.17	0.08	0.10	0.16	0.14	0.00	0.00	0.16
re3d	0.18	0.20	0.15	0.14	**0**.**48**	0.04	0.11	0.06	0.14	0.09	0.00	0.00	0.13
i2b2-06	0.10	0.09	0.16	0.11	0.00	**0**.**74**	0.03	0.03	0.06	0.01	0.00	0.00	0.11
ontonotes	0.19	0.10	0.10	0.03	0.11	0.06	**0**.**30**	0.04	0.08	0.03	0.00	0.00	0.09
SEC	0.04	0.03	0.03	0.04	0.00	0.00	0.03	**0**.**84**	0.02	0.01	0.00	0.00	0.09
WNUT17	0.12	0.09	0.08	0.12	0.05	0.09	0.03	0.01	**0**.**18**	0.03	0.00	0.00	0.07
GUM	0.09	0.10	0.07	0.11	0.06	0.02	0.03	0.02	0.06	**0**.**24**	0.00	0.00	0.07
sciERC	0.00	0.00	0.00	0.00	0.00	0.00	0.00	0.00	0.00	0.00	**0**.**28**	0.00	0.02
AnEM	0.00	0.00	0.00	0.00	0.00	0.00	0.00	0.00	0.00	0.00	0.00	**0**.**22**	0.02

Fine-tuning uses the BioBERT-base model

**Table 16:** F1 scores on full-sized datasets

	conll	cerec	ontonotes	i2b2-06	wikigold	WNUT17	GUM	re3d	SEC	BTC	sciERC	AnEM	average f1
conll	**0**.**88**	0.32	0.26	0.11	0.58	0.21	0.10	0.29	0.36	0.23	0.00	0.00	**0**.**28**
cerec	0.30	**0**.**87**	0.11	0.06	0.30	0.12	0.20	0.20	0.31	0.15	0.00	0.00	0.25
ontonotes	0.23	0.14	**0**.**92**	0.10	0.22	0.12	0.07	0.15	0.07	0.15	0.00	0.00	0.23
i2b2-06	0.33	0.22	0.17	**1**.**00**	0.36	0.09	0.09	0.26	0.23	0.16	0.00	0.00	0.23
wikigold	0.49	0.25	0.29	0.10	**0**.**84**	0.11	0.11	0.28	0.38	0.15	0.00	0.00	0.21
WNUT17	0.40	0.24	0.25	0.14	0.43	**0**.**61**	0.09	0.23	0.19	0.25	0.00	0.00	0.21
GUM	0.15	0.16	0.04	0.03	0.21	0.03	**0**.**63**	0.26	0.04	0.06	0.00	0.00	0.21
re3d	0.15	0.21	0.11	0.03	0.16	0.07	0.13	**0**.**53**	0.09	0.11	0.00	0.00	0.20
SEC	0.21	0.19	0.15	0.11	0.18	0.08	0.05	0.10	**0**.**90**	0.21	0.00	0.00	0.20
BTC	0.50	0.33	0.32	0.14	0.58	0.22	0.12	0.30	0.31	**0**.**85**	0.00	0.00	0.20
sciERC	0.00	0.00	0.00	0.00	0.00	0.00	0.00	0.00	0.00	0.00	**0**.**55**	0.00	0.19
AnEM	0.00	0.00	0.00	0.00	0.00	0.00	0.00	0.00	0.00	0.00	0.00	**0**.**77**	0.19

Fine-tuning uses the BioBERT-base model

## Footnotes

1 We use only the English data.

2 Similiar to CONLL, only English data is used.

3 This approach is inspired by the methods used to address the divide by zero problem in multi-class logistic regression in machine learning.

4
https://www.elsevier.com/authors/policies-and-guidelines/credit-author-statement
